# Supplementary material for: The human coronavirus HCoV-229E S-protein structure and receptor binding
Source: eLife. 2019 Oct 25;8:e51230. doi: 10.7554/eLife.51230 (PMC6970540; doi:10.7554/eLife.51230)
Supplement: Supplementary file 1. [file elife-51230-supp1.docx]

**Supplementary file 1. Cryo-EM data collection and refinement statistics.**

| **Data Collection** |  |
| --- | --- |
| Electron Microscope | Titan Krios G3 |
| Camera | Falcon 3EC |
| Voltage (kV) | 300 |
| Nominal Magnification | 75,000 |
| Calibrated physical pixel size (Å) | 1.06 |
| Total exposure (e/Å2) | 42.7 |
| Exposure rate (e/pixel/s) | 0.8 |
| Number of frames | 30 |
| Defocus range (µm) | 1.8 to 2.2 |
|  |  |
| **Image Processing** |  |
| Motion correction software | cryoSPARC v2, MontionCor2 |
| CTF estimation software | CTFFIND4,GCTF |
| Particle selection software | cryoSPARC v2, RELION |
| Micrographs used | 3600 |
| 3D map classification and refinement software | cryoSPARC v2, RELION |
|  |  |
| **Model building** |  |
| Modeling software | COOT, ROSETTA, PHENIX, ISOLDE |
| Particle images contributing to maps | 71,350 |
| Applied symmetry | C3 |
| Global resolution (FSC = 0.143, Å) | 3.1 |
| RMS bond length (Å) | 0.012 |
| RMS bond angle (°) | 1.77 |
| Ramachandaran outliers (%) | 0.0 |
| Ramachandran favoured (%) | 95.72 |
| Clashscore | 0.24 |
| MolProbity score | 0.89 |
| EMRinger score | 3.81 |
| Protein residue number | 2895 |
| Ligands |  |
| BMA | 6 |
| NAG | 63 |
| MAN | 12 |
