# Supplementary material for: The human coronavirus HCoV-229E S-protein structure and receptor binding
Source: eLife. 2019 Oct 25;8:e51230. doi: 10.7554/eLife.51230 (PMC6970540; doi:10.7554/eLife.51230)
Supplement: Supplementary file 2. — **Values in parentheses are for the highest-resolution shell. [file elife-51230-supp2.docx]

**Supplementary file 2. X-ray Crystallographic data collection and refinement statistics. ***

|  | Class III RBD | Class IV RBD | Class V RBD |
| --- | --- | --- | --- |
| Wavelength(Å) | 0.9795 | 0.9795 | 1.033 |
| Resolution range | 50.0-3.00 (3.11 -3.00) | 50.0-2.75 (2.85-2.75) | 50.0-2.35 (2.43-2.35) |
| Space group | P 1 21 1 | P 1 21 1 | P 1 21 1 |
| Unit cell a,b,c | 99.32 98.52 153.62 | 99.16 98.57 147.78 | 99.56 98.78 147.53 |
| Unit cell α,β,γ | 90 104.4 90 | 90 104.4 90 | 90 104.6 90 |
| Total reflections | 209783 (22013) | 272415 (27367) | 405628 (33927) |
| Unique reflections | 57625 (5712) | 71808 (7122) | 114585 (8296) |
| Multiplicity | 3.6 (3.9) | 3.8 (3.8) | 3.5 (3.1) |
| Completeness (%) | 99.77 (99.72) | 99.84 (99.89) | 96.01 (72.51) |
| Mean I/sigma(I) | 11.20 (2.49) | 16.18 (3.87) | 14.01 (2.99) |
| Wilson B-factor | 85.7 | 49.0 | 30.0 |
| R-merge | 0.070 (0.587) | 0.068(0.423) | 0.052 (0.377) |
| R-meas | 0.082 (0.683) | 0.080 (0.492) | 0.062 (0.459) |
| R-pim | 0.043 (0.348) | 0.041 (0.250) | 0.032 (0.258) |
| CC1/2 | 0.998 (0.881) | 0.997 (0.908) | 0.995 (0.872) |
| CC* | 0.999 (0.968) | 0.999 (0.975) | 0.999 (0.965) |
| Reflections used in refinement | 57590 (5704) | 71791 (7121) | 110682 (8296) |
| Reflections used for R-free | 2881 (286) | 3591 (357) | 5552 (401) |
| R-work | 0.205(0.301) | 0.192 (0.263) | 0.181 (0.231) |
| R-free | 0.240 (0.357) | 0.224 (0.294) | 0.220 (0.303) |
| CC(work) | 0.948 (0.831) | 0.948 (0.871) | 0.949 (0.861) |
| CC(free) | 0.925 (0.648) | 0.919 (0.783) | 0.926 (0.740) |
| Number of non-hydrogen atoms | 16543 | 16575 | 17357 |
| macromolecule atoms | 16234 | 16023 | 16260 |
| ligand atoms | 304 | 324 | 296 |
| solvent atoms | 5 | 228 | 801 |
| Protein residues | 2030 | 1993 | 2029 |
| RMS bonds (Å) | 0.003 | 0.005 | 0.005 |
| RMS angles (°) | 0.53 | 0.89 | 0.64 |
| Ramachandran favored (%) | 96.18 | 97.82 | 97.61 |
| Ramachandran allowed (%) | 3.77 | 2.18 | 2.39 |
| Ramachandran outliers (%) | 0.05 | 0 | 0 |
| Rotamer outliers (%) | 0.06 | 1.19 | 0.34 |
| Clashscore | 2.89 | 2.74 | 1.88 |
| Average B-factor | 97.74 | 50.03 | 39.46 |
| macromolecules | 97.24 | 52.70 | 39.12 |
| ligands | 124.91 | 76.06 | 61.76 |
| solvent | 55.76 | 44.04 | 37.99 |

*Values in parentheses are for the highest-resolution shell.
